# N6-methyladenosine RNA methylation, a new hallmark of metabolic reprogramming in the immune microenvironment

**DOI:** 10.3389/fimmu.2024.1464042

**Published:** 2024-12-20

**Authors:** Xiaoyue Li, Lin Peng, Xuelian Yang, Jing Luo, Jianmei Wang, Kelin Mou, Huan Zhou, Yuhao Luo, Li Xiang

**Affiliations:** ^1^ Department of Oncology, The Affiliated Hospital of Southwest Medical University, Luzhou, China; ^2^ School of Life Sciences, Yunnan University, Kunming, China; ^3^ Department of Bone and Joint, The Affiliated Hospital of Southwest Medical University, Luzhou, China; ^4^ Department of Cardiology, The Affiliated Hospital of Southwest Medical University, Luzhou, China; ^5^ Department of Pathology, The Affiliated Hospital of Southwest Medical University, Luzhou, China

**Keywords:** N6-methyladenosine, tumor microenvironment, metabolic reprogramming, immunotherapy, m6A modification

## Abstract

N6-methyladenosine is one of the most common and reversible post-transcriptional modifications in eukaryotes, and it is involved in alternative splicing and RNA transcription, degradation, and translation. It is well known that cancer cells acquire energy through metabolic reprogramming to exhibit various biological behaviors. Moreover, numerous studies have demonstrated that m6A induces cancer metabolic reprogramming by regulating the expression of core metabolic genes or by activating metabolic signaling pathways. Meanwhile, m6A modifications and related regulators are key targets in the regulation of immune effects. We further summarize how m6A modifications contribute to tumor metabolism, and how these events affect the tumor immune microenvironment, with a specific focus on different cell types. Finally, we focus on the specific applications of this field to tumor immunotherapy. We review the potential role of m6A in metabolic reprogramming of tumor immune microenvironment and its regulatory mechanism, with the aim of providing new targets for tumor metabolic regulation and immunotherapy.

## Background

The most common internal RNA modifications include N6-adenylate methylation (m6A), N1-adenylate methylation (m1A), and cytosine hydroxylation (m5C) (1). m6A is the most common post-transcriptional modification of eukaryotic mRNAs, accounting for more than 60% of RNA methylation modifications (2). Internal mRNA modifications are used to maintain mRNA stability (3). In the 1970s, Desrosiers et al. identified m6A in the mRNA of human mammalian cells; however, its function and mechanism remain largely unexplored (4). It was not until 2011, when He et al. reported the significant role of N6-methyladenosine in nuclear RNA and discovered that this comprises a reversible modification, that studies on N6-methyladenosine were initiated (5).

Prior to 2012, no research had been performed to identify m6A modifications at the genome-wide or transcriptome levels. Meyer and Dominissini reported the first large-scale high-throughput assessment of m6A methylation in humans and mice at the transcriptional level (6, 7). In 2015, Linder et al. were the first to study m6A at the single-base level (8). Subsequent studies confirmed the critical role of m6A methylation in various physiological functions in the human body, including hematopoiesis (9), central nervous system regulation (10, 11), and reproduction (12). Moreover, m6A plays an important regulatory role in the occurrence and development of various human diseases such as Alzheimer’s disease (13), myocardial hypertrophy (14), and diabetes (15). Notably, it also plays a vital role in regulating the biological behavior of tumors (16).

One of the most important characteristics of the body is active energy metabolism, which involves continuous energy intake and consumption (17). The primary source of energy for the body is the metabolism of three major nutrients, glucose, lipids, and proteins (18). Stable metabolism is essential for maintaining normal body functions, especially under pathological conditions. The tumor immune microenvironment refers to the complex regulatory network occurring in the tumor microenvironment (TME), which includes various immune cells, cytokines, and fibroblasts, and this has a pivotal role in tumor initiation, progression, metastasis, and the response to therapy (19). Normal metabolism is vital for timely and effective immune responses (20, 21); Meanwhile, when human immunity is downregulated due to internal environmental dysfunction or disease, the immune system is activated. The immune system of the body recognizes self and foreign substances and eliminates antigenic foreign bodies through immune responses, thus maintaining a physiological balance (22).

However, immune activation leads to dynamic changes in immune cells, such as the rapid growth and proliferation of activated T cells, which require a substantial energy supply to support normal immune functions (23). Providing sufficient biomolecules (lipids, amino acids, and nucleotides) to synthesize new cellular components is key to supporting these cellular activities (24, 25). Additionally, abnormal metabolism or the presence of tumors can lead to the production of abnormal metabolites, such as lactic acid, altering the physical and chemical properties of the immune microenvironment and affecting the normal immune responses of immune cells (26). Studies have shown that High cholesterol levels can affect the effectiveness of immune checkpoint inhibitors in advanced cancers (27). Thus, the regulation of metabolism is crucial for an effective immune response. Interestingly, some researchers have found that immune cell metabolism can also influence anti-tumor immune efficacy (28, 29). Therefore, exploring the relationship between the immune response and metabolic regulation is of great importance. However, the specific role of m6A in the metabolic reprogramming of the tumor-induced immune microenvironment and the associated mechanisms remain unclear.

This paper reviews the role of m6A modifications in the immune microenvironment and metabolic regulation, as well as the crosstalk between m6A and these processes. In addition, potential applications and possible future directions in this field are discussed.

## N6-methyladenosine regulators

**Figure 1:**
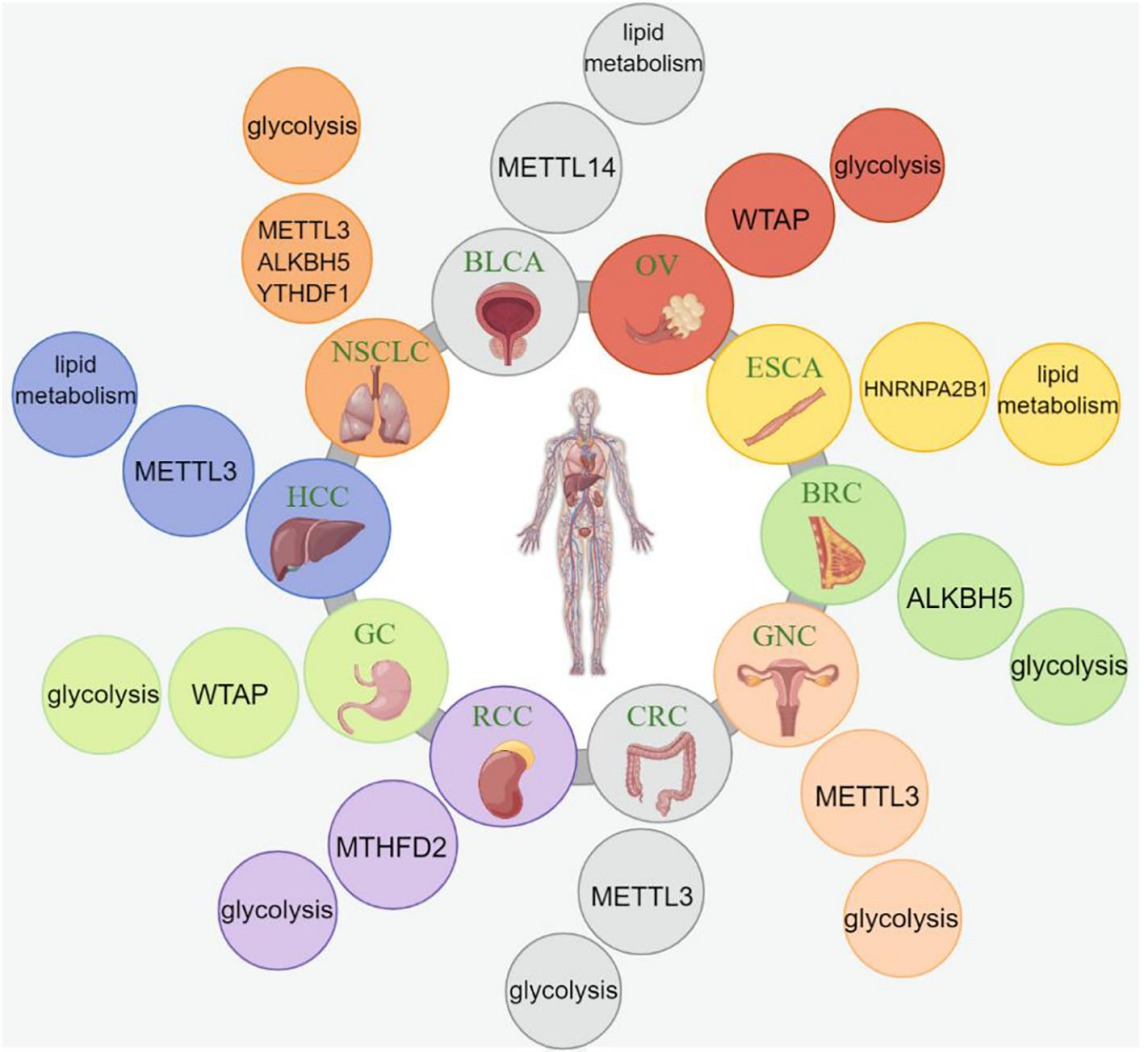
The process and functions of m6A methylation. m6A methylation is catalyzed by the writer complex including METTL3, METTL14, WTAP, RBM15/15B, CBLL1, and ZC3H13. The m6A modification is erased by demethylases including FTO, ALKBH10, ALKBH3 and ALKBH5. The m6A-modified RNA reader proteins include YTHDF1/2/3, YTHDC1/2, IGF2BP1/2/3, and eIF3. m6A modification modulates the regulation of gene expression, RNA splicing, mRNA stability, miRNA biogenesis, m6A switch, RNA translocation, and RNA decay.

The m6A modification, comprising N6-methyladenosine, refers to the methylation of the sixth nitrogen (N) atom of adenylate (A) in RNA. RNA modifications are reversible and tightly regulated by three essential types of proteins, namely methyltransferases (writers), demethylases (erasers), and m6A readers (30). An overview of m6A modifications during the mRNA life cycle is shown in **Figure 1**.

### Writers

Methyltransferases, including methyltransferase-like 3 and 14 proteins (METTL3 and METTL14, respectively) and their cofactors, such as WT1-associated protein (WTAP), Vir-like m6A methyltransferase associated (VIRMA), zinc finger CCCH-type containing 13 (ZC3H13), and RNA-binding motif protein 15/15B (RBM15/15B), play a crucial role in the composition of the methyltransferase complex (MTC), thereby catalyzing the modification of adenosine on mRNA to form m6A (31, 32). METTL3, METTL14, and WTAP are the core proteins of m6A methyltransferase, and they function together to form the MTC (33). In 1997, Joseph et al. discovered METTL3 as the most important and first known component of this complex (34, 35). Interestingly, METTL16, which shares homology with METTL3, has been identified as a novel, independent RNA methyltransferase that regulates cellular S-adenosylmethionine levels and methylates U6 small nuclear RNA (36). One study found that platelet-derived growth factor signaling impedes mitophagy in glioblastoma stem cells by regulating N6-methyladenosine, thereby potentially contributing to the tumorigenic properties and survival of these cells (37).This intricate network of methyltransferases and their associated proteins highlights their essential role in mRNA modifications, which are critical for various cellular processes and could be a potential target for therapeutic interventions in tumors.

### Erasers

Demethylases, such as fat mass and obesity-associated protein (FTO) and alkB homolog 5 (ALKBH5), play crucial roles in demethylating bases that undergo m6A modifications (38). FTO was the first protein discovered to catalyze the demethylation of m6A, and it collaborates with ALKBH5 to regulate m6A levels in the transcriptome (5, 39, 40). The demethylation function of ALKBH5, the second demethylase identified after FTO, tends to be targeted toward tRNA, rather than mRNA or rRNA (41). ALKBH5 has been implicated in the regulation of lncRNAs, cancer stem cells, autophagy, and hypoxia (42). Lv D et al. conducted that the epidermal growth factor receptor (EGFR) promotes the nuclear retention of ALKBH5 in glioblastoma. This retention leads to a reduction in m6A levels, thereby protecting glioblastoma cells against ferroptosis, a form of regulated cell death involving iron-dependent lipid peroxidation (43). Moreover, this protein was found to modify PD-L1 via mRNA epigenetics, suggesting its potential role in immunotherapy responses, which could provide new insights into cancer immunotherapy (44). The study of demethylases and their roles in m6A modifications and transcriptome regulation provides exciting possibilities for understanding the epigenetic mechanisms involved in various cellular processes, including cancer development and immunotherapy. Further research in this area could reveal novel therapeutic targets and treatment strategies for cancer and other related diseases.

### Readers

m6A-modified RNA requires specific m6A-binding proteins for recognition to perform specific biological functions. These proteins are known as m6A readers (45). Among m6A readers, the YT521-B homology (YTH) domain family includes YTHDC1, YTHDC2, YTHDF1, YTHDF2, and YTHDF3, which has a conserved m6A binding domain (46). Heterogeneous nuclear ribonucleoprotein family proteins (HNRNPs) and eukaryotic initiation factor 3 (eIF3) are the key m6A readers (47). The functions of these m6A reader proteins mainly involve specific binding to the m6A methylation region on RNA, weakening homologous binding to RNA-binding proteins, and altering the RNA secondary structure to modulate protein–RNA interactions (48). These interactions between m6A readers and modified RNA play crucial roles in various cellular processes, including the regulation of gene expression, RNA splicing, mRNA stability, and translation. Accordingly, understanding the mechanisms of m6A reader proteins is essential to elucidate the epigenetic regulatory networks governed by m6A modifications and their impact on cellular functions and disease development.

## Tumor metabolic reprogramming

In recent years, a substantial body of research has focused on the phenomenon by which cancer cells undergo anaerobic glycolysis, producing large amounts of lactic acid, even in the presence of oxygen, to sustain their metabolic needs. This phenomenon, known as the Warburg effect (49), has triggered a surge in metabolic research. Furthermore, studies have revealed that tumor nutrition is not limited to glucose alone and that cancer cells also utilize unconventional sources of nutrients. Additionally, the interplay between cancer cell metabolic alterations and the TME, involving various host immune cells and microbiota, has been identified as a key mechanism in the regulation of cancer progression (50). Moreover, abnormal metabolite accumulation, impaired nutrient clearance mechanisms, and autonomous changes in metabolic pathway flux contribute to tumor growth and survival.

In addition to extensive research on mechanisms through which the Warburg effect promotes tumor growth, the tricarboxylic acid cycle (TCA) serves as a crucial anabolic hub supporting tumor growth (51, 52). Thus, it can be concluded that both glycolysis and the TCA cycle play significant roles in supporting tumor growth through metabolite biosynthesis. Moreover, the synthesis and breakdown of fatty and amino acids, particularly glutamine, serine, and glycine, are critical for the metabolic regulation of tumor development. However, numerous aspects of tumor-related metabolic reprogramming require further exploration. This section reviews the regulation of these three metabolic pathways and the associated research progress in cancer treatment strategies. Understanding the intricate metabolic alterations in cancer cells and their interactions with the tumor microenvironment could provide a foundation for novel therapeutic approaches to combat cancer.

### Glucose metabolism

Glucose metabolism plays a crucial role in overall metabolic processes, as it pertains to one of the three major energy substrates in the body. Glycolysis is the primary pathway of glucose metabolism, and its end product, pyruvate, can be converted to lactic acid in the absence of oxygen or can enter the TCA cycle for oxidative phosphorylation. Despite producing a lower amount of ATP than aerobic oxidation, glycolysis has a much faster ATP production rate, allowing for the generation of many metabolites to provide energy for tumor initiation and development. This explains why tumors often utilize anaerobic glycolysis even in the presence of ample oxygen. Consequently, targeting glycolysis has emerged as a potential strategy for tumor therapy.

Under pathological conditions, cancer cells increase glucose uptake, making it possible to detect tumors using positron emission tomography and evaluate the tumor response (53). Key enzymes involved in glucose metabolism and their related target genes can regulate metabolic processes, thereby influencing tumor growth characteristics. For example, hexokinase II (HKII), aldehyde-3-phosphate dehydrogenase (GAPDH), the pyruvate kinase (PK)-M2 isoform, and lactate dehydrogenase (LDH) play crucial roles in regulating glucose metabolism. In addition, the cisplatin-induced p53-mediated downregulation of HKII expression affects chemotherapy sensitivity in ovarian cancer (54). Moreover, GAPDH plays a vital role in tumor cell survival, angiogenesis, and the post-transcriptional regulation of tumor cell mRNA. Additionally, LDHA phosphorylation and activation can promote cancer cell invasion and metastasis, making it a potential therapeutic target and prognostic marker for human cancers (55). The Fibrillin-1/VEGFR2/STAT2 signaling axis enhances chemoresistance in ovarian cancer by regulating glycolysis and angiogenesis within both organoids and cancer cells (56).The study by Zhen Y et al. suggests that oncogenic Fibroblast Growth Factor Receptor 2(FGFR2) signaling drives NF-κB-dependent glycolysis in intrahepatic cholangiocarcinoma and that metabolic reprogramming in response to FGFR inhibition confers new targetable vulnerabilities (57). Understanding the intricate mechanisms underlying glucose metabolism and the associated enzymes in cancer cells provides valuable insights into potential therapeutic targets for cancer treatment.

### Lipid metabolism

Lipid metabolism can be divided into two main processes, anabolism and catabolism. In cancer, tumor cells exhibit altered lipid metabolism to meet specific needs. For example, as tumor cells proliferate, they increase fatty acid synthesis to support cell growth and reproduction. Fatty acids play crucial roles in energy storage, cell membrane synthesis, and signaling molecule production (25).

Fatty acid β-oxidation is a significant pathway involved in fat breakdown, and tumor cells utilize the energy generated through this process to fuel their biological activities, including proliferation and invasion. The proper regulation of lipid metabolism, including fatty acid uptake, hydrolysis, and synthesis, is essential for maintaining cellular homeostasis (58). Energy stored in fat primarily exists in the form of fatty acids, which are taken up by tissues through various transporters, such as cluster of differentiation 36 (CD36), the FA transport protein (FATP) family, and plasma membrane fatty acid-binding proteins (FABPs) (59). Notably, blocking CD36 with neutralizing antibodies has been shown to inhibit oral cancer metastasis (60).

Studies have demonstrated that lipid metabolism has a significant effect on tumor progression and resistance to therapy. For example, Wang et al. found that inhibiting the JAK/STAT3 pathway could regulate breast cancer self-renewal and the expression of genes related to lipid metabolism, ultimately affecting fatty acid oxidation and chemotherapy resistance (61). Similarly, in acute myeloid leukemia (AML) and ovarian cancer, the overexpression of fatty acid-β-oxidation-related enzymes, such as CPT1A, has been associated with poor patient prognosis (62). Moreover, in various cancer types, the inhibition of CPT1 significantly inhibits cell growth and viability (63, 64).Moreover, oncogene amplification in growth factor signaling pathways drives cancer cells to depend on membrane lipid remodeling for proper function (65). This dependency suggests new targets for therapeutic intervention in cancer treatment. These findings highlight the importance of lipid metabolism in tumor biology and provide potential targets for cancer therapy. Understanding the intricate regulation of lipid metabolism in cancer cells could lead to the development of novel therapeutic approaches and strategies to combat cancer progression and enhance treatment efficacy.

### Amino acid metabolism

Increasing studies have revealed that amino acids, such as glutamine, serine, and glycine, are not only substrates for protein synthesis but also important metabolites and metabolic regulators that support cancer cell growth. Among these amino acids, glutamine is a key metabolic regulator of cancer and is involved in various pathways, including energy metabolism, signal transduction, and the biosynthesis of cancer cells (66). Son et al. reported that in pancreatic cancer, oncogenic KRAS regulates glutamine metabolic reprogramming by transcriptionally upregulating aspartate aminotransferase (AST) expression and inhibiting glutamate dehydrogenase 1 (GLUD1) (67). Additionally, the absence of liver kinase B1 (LKB1) was found to lead to the induction of a growth-promoting metabolic program in proliferating cells. Cells lacking LKB1 exhibit the increased uptake and utilization of glucose and glutamine, which supports elevated cellular ATP levels and increased macromolecule biosynthesis (68). In the context of T cell acute lymphoblastic leukemia, the inhibition of serine hydroxymethyltransferase (SHMT), a key enzyme involved in serine metabolism, has shown a synergistic anti-tumor effect with methotrexate (69). Similarly, targeting SHMT revealed a specific metabolic vulnerability in diffuse large B cell lymphoma (70). These findings underscore the significance of amino acids, especially glutamine, serine, and glycine, as vital components of cancer cell metabolism and as potential targets for therapeutic interventions.

### Nucleotide metabolism

Nucleotide metabolism plays a critical role in various biological processes, including DNA replication, repair, and cellular signaling. Aberrant nucleotide metabolism has been implicated in the development and progression of various tumors (71). Nucleotide metabolism involves the synthesis, breakdown, and recycling of nucleotides, which are essential for the maintenance of cellular functions (72, 73). In tumor cells, changes in nucleotide metabolism can lead to genomic instability, cell proliferation and resistance to anticancer therapies. Targeting nucleotide metabolism, especially in tumor immunotherapy, is a promising new direction to enhance immune efficacy (74).

Nucleotide synthesis occurs through two main pathways: *de novo* synthesis and salvage pathways. Several key enzymes and pathways in nucleotide metabolism have been identified to play a crucial role in tumor development and progression.High levels of hypoxanthine-guanine phosphoribosyltransferase (HPRT), an enzyme involved in the salvage pathway, have been detected in lung cancer cells. This contributes to the increased synthesis of nucleotides, which are essential for rapid cellular proliferation. Targeting HPRT may offer a novel therapeutic approach for treating lung cancer (75).It has been highlighted that blocking equilibrative nucleoside transporter 1 with CNX-774 can overcome resistance to dihydroorotate dehydrogenase inhibition, offering a promising approach to treating pancreatic cancer that has become resistant to current therapeutic strategies (76).Nathanson DA et al. explored the strategy of simultaneously targeting multiple convergent nucleotide biosynthetic pathways, which could be a potent approach for eradicating leukemia by exploiting the vulnerabilities in cancer cells’ nucleotide synthesis, thereby enhancing the effectiveness of treatment (77). Surprisingly, Keshet R et al. reveal that the suppression of purine synthesis not only raises the pyrimidine to purine ratio but also boosts the expression of the immunoproteasome. Consequently, this leads to a notable augmentation in the responsiveness of autologous primary CD8+T cells to anti-PD-1 treatment. The findings imply that patients with high-Argininosuccinate synthase1 expressing cancers could derive advantage from purine synthesis inhibitors, potentially making them more receptive to immune checkpoint blockage therapies (78).Therefore, targeting nucleotide metabolism holds promise for enhancing cancer treatment by contributing to genomic stability, overcoming drug resistance, and improving immune therapy efficacy.

## Metabolic regulation of the immune microenvironment

Metabolism in non-cancer cells within the tumor microenvironment, including endothelial, fibroblast, and immune cells, plays a critical role in regulating tumor progression (50). Among these cells, immune cells are particularly important for immune activation and tumor evasion, and they require unique metabolic pathways to maintain their normal functions. Studies have shown that dendritic cells (DCs) and T cells undergo changes in glucose and amino acid metabolism upon activation (79). For example, CD8^+^ T cells upregulate the expression of cytosolic phosphoenolpyruvate carboxykinase (PCK1), a key regulator of glycolysis, tricarboxylic acid cycle, and gluconeogenesis, to increase glycogenesis via gluconeogenesis. These findings indicate that the activities of immune cells are tightly regulated by their metabolism and that metabolic reprogramming plays a crucial role in tumor growth and differentiation, affecting the functions of tumor-related immune cells.

Increased glycolysis can lead to lactic acid accumulation in the tumor microenvironment, impairing CD8^+^ T-cell proliferation, cytokine production, and cytolytic activity (80). Additionally, L-arginine metabolism affects the differentiation and survival of activated human T cells. Moreover, L-arginine restriction inhibits human T-cell differentiation, whereas increased intracellular L-arginine levels enhance mouse T-cell survival, both *in vitro* and *in vivo* (81). Wang et al. demonstrated that lipid uptake through CD36 and PPAR-β signaling promotes mitochondrial adaptation in Treg cells. Further, enhancing lipid uptake and metabolism supports the inhibitory activity and survival of Treg cells within tumors, suggesting that targeting the metabolic regulation of Treg cells is an attractive anti-tumor strategy (82). Thus, targeting tumor metabolic pathways is a promising approach for enhancing tumor immunotherapy. By understanding and manipulating the metabolism of tumor-related immune cells, we can improve the efficacy of cancer immunotherapies and develop novel strategies for combating cancer.

## Role of m6A modifications in tumor metabolism

Metabolic reprogramming is a hallmark of malignant tumor cells. Metabolism can be broadly categorized into two processes, synthesis and decomposition. Tumor cells require metabolism not only for life-sustaining synthesis processes but also for providing energy for vital activities, such as tumorigenesis, proliferation, and migration (83). Notably, studies have demonstrated that m6A regulation participates extensively in the metabolic reprogramming of tumors (84). In this section, we present a comprehensive review of the regulatory roles of m6A regulators in tumor metabolism and the associated mechanisms.

**Figure 2:**
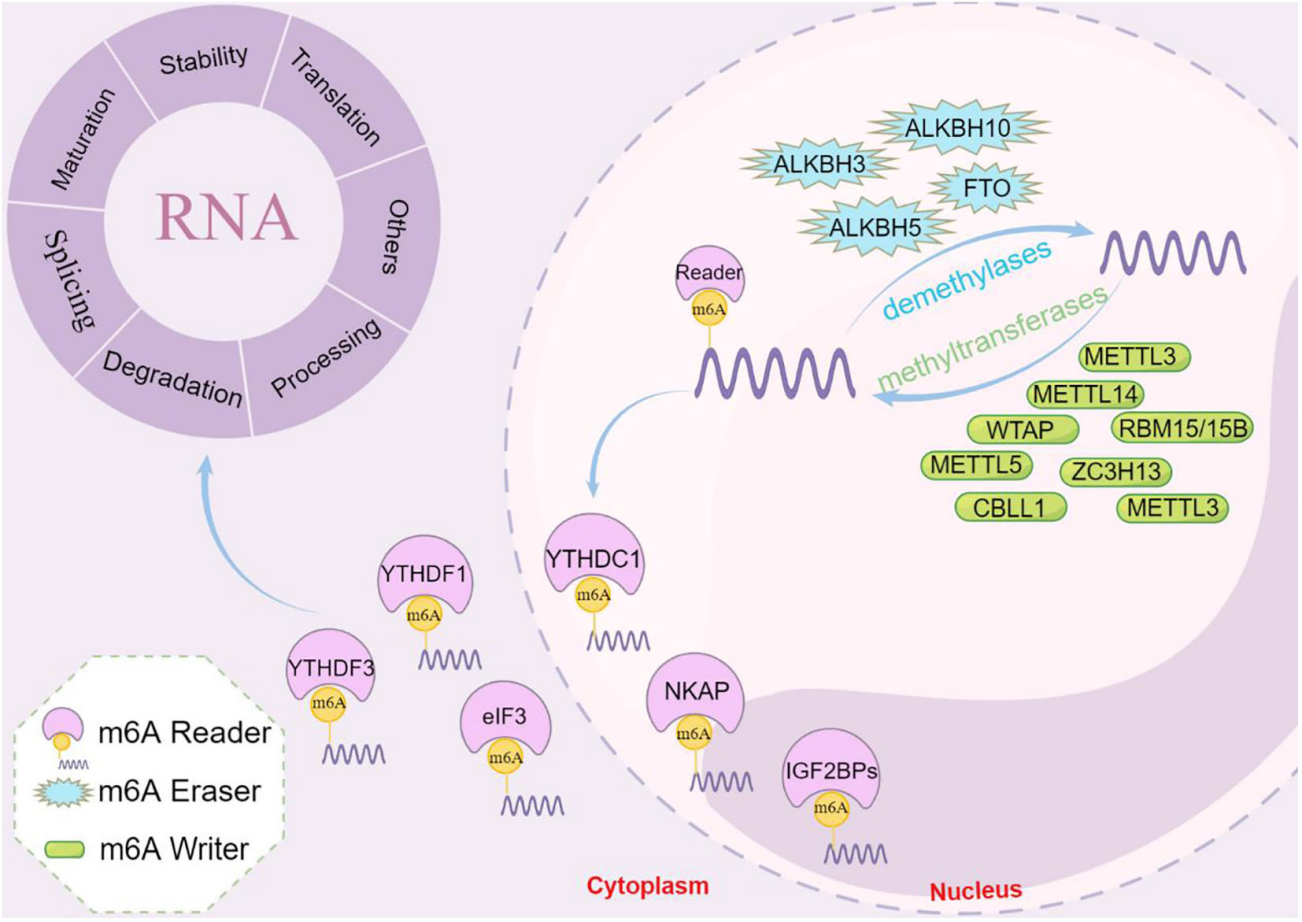
Schematic diagram of the regulation of m6A regulators with respect to tumor metabolism in each system. In OV and GC, WTAP further mediates tumorigenesis and progression by regulating glycolysis. Similarly, in HCC, METTL3 regulates lipid metabolism and thus affects tumor progression. In BLCR, METTL14 regulates lipid metabolism and further influences tumor life activities. Therefore, in different tumors, the tumor metabolism is further mediated by regulating the corresponding m6A regulators, which ultimately affects the life activities of tumors.

**Figure 3:**
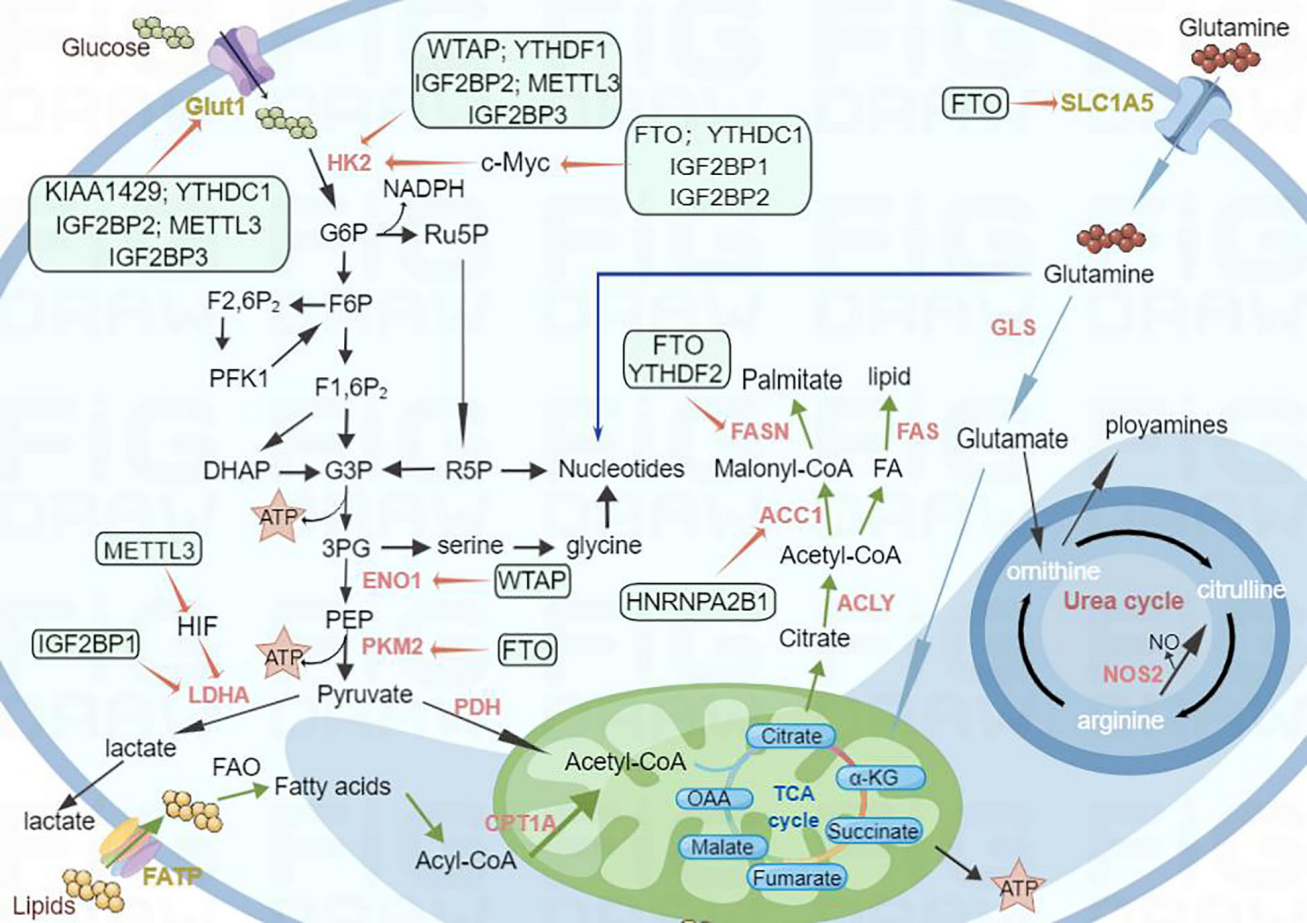
Schematic diagram of the regulatory mechanism of m6A modifiers with respect to tumor metabolism. Metabolic reprogramming in cancer cells is a complex and dynamic process, and the m6A modification of RNA has emerged as a significant factor that orchestrates these metabolic changes. For example, m6A regulators such as WTAP and YTHDF1 mediate glycolysis by regulating HK2, a key enzyme in glucose metabolism. FTO and YTHDF2 affect lipid metabolism by regulating FASN; similarly, FTO affects glutamine metabolism by regulating SLC1A5. Thus, m6A regulators have a significant effect on the regulation of metabolic changes.

Metabolic reprogramming in cancer cells is a complex and dynamic process, and the m6A modification of RNA has emerged as a significant factor that orchestrates these metabolic changes. Understanding the intricate interplay between m6A regulators and tumor metabolism could provide new options for targeted therapies and the development of novel cancer treatment strategies. The roles of m6A modifications in tumor metabolism are summarized in **Table 1**. The regulation of m6A regulators in each system pertaining to tumor metabolism is depicted in **Figure 2**, and the regulatory mechanism of m6A modifiers in tumor metabolism is shown in **Figure 3**.

**Table 1:** Role of m6A modifications in tumor metabolism.

System	m6A regulators	metabolism	Mechanisms	Ref.
NSCLC	METTL3↑ALKBH5↓YTHDF1↑	Glycolysis↑	Dysregulation of METTL3, ALKBH5, and YTHDF1-mediated ENO1 translation	(85)
ESCA	HNRNPA2B1↑	Lipid metabolism↑	Upregulation of fatty acid synthase ACLY and ACC1 expression	(86)
ESCA	METTL3↑YTHDF	Aerobic glycolysis↑	METTL3 recruits YTHDF to degrade APC mRNA, which further leads to increased β-catenin and β-catenin-mediated cyclin D1, c-Myc, and PKM2 expression	(87)
GC	WTAP↑	Glycolysis↑	Enhances the stability of HK2 mRNA by binding to the 3′-UTR m6A site	(88)
GC	KIAA1429↑	Aerobic glycolysis↑	KIAA1429 catalyzes LINC00958, which further enhances the stability of GLUT1 mRNA transcripts	(89)
HCC	METTL3↑	Lipogenesis↑	METTL3 regulates the m6A modification of LINC00958	(90)
CRC	METTL3↑	Glycolysis↑	METTL3 promotes the stability and activation of HK2 and GLUT1 and activates glycolytic pathways	(91)
CRC	IMP2↑	Aerobic glycolysis↑	IMP2 activates the Warburg effect by stabilizing the ZFAS1/OLA1 axis	(92)
CxCa	METTL3↑	Aerobic glycolysis↑	METTL3 enhances the stability of HK2 through YTHDF1-mediated m6A modification	(93)
CxCa	ALKBH5↑	Lipid metabolism↓	The ALKBH5/SIRT3/ACC1 axis inhibits tumor growth, lipid metabolism, and tumorigenesis	(94)
CxCa	IGF2BP2↓	Glycolysis↓	E6/E7 protein regulates Myc mRNA m6A modification through IGF2BP2	(95)
OV	WTAP↑	Aerobic glycolysis↑	WTAP interacts with DGCR8 to regulate miR-200 expression	(96)
PRAD	METTL3↑	Glycolysis↑	METTL3 promotes the stability of lncRNA SNHG7 through m6A modification	(97)
RCC	METTL14↓	Glycolysis↑	The METTL14/BPTF axis regulates epigenetic and metabolic remodeling in renal cell carcinoma	(98)
RCC	IGF2BP1↑	Aerobic glycolysis↑	IGF2BP1 recognizes the m6A modification site on LDHA mRNA and enhances its mRNA stability	(99)
RCC	MTHFD2↑	Glycolysis↑	MTHFD2 could promote the translation of HIF-2α	(100)
BLCA	ALKBH5↓	Glycolysis↑	ALKBH5 mediates the stability of casein kinase 2α and further regulates the glycolytic pathway	(101)
BLCA	METTL14↑	Lipid metabolism↑	The METTL14/lncDBET/FABP5 axis regulates lipid metabolism	(102)

NSCLC, non small cell lung cancer; ESCA, esophageal carcinoma; GC, gastric carcinoma; HCC, hepatocellular carcinoma; CRC, colorectal cancer; CxCa, Cervical Cancer; OV, ovarian cancer; PRAD, prostate adenocarcinoma; RCC, renal cell carcinoma; BLCA, bladder urothelial carcinoma.

↑ indicates that this metabolic process is enhanced, and the ↓ indicates that this metabolic process is weakened.

### Respiratory system

Respiratory system tumors include various malignancies, such as nasopharyngeal, lung, and laryngeal cancers. Non-small cell lung cancer (NSCLC) is associated with the highest mortality rate worldwide. Research has revealed that m6A RNA methylation plays a critical role in NSCLC progression by regulating mitochondrial RNA processing endonuclease (RMRP) stability and affecting the TGFBR1/SMAD2/SMAD3 pathway (103). Ma et al. showed that the dysregulation of METTL3, ALKBH5, and YTHDF1-mediated ENO1 translation creates a favorable metabolic environment in lung adenocarcinoma cells, promoting glycolysis and facilitating tumor occurrence and progression (85).

Further, the dysregulation of this pathway contributes to cancer progression and metastasis in NSCLC, which is associated with a high mortality rate worldwide. Li et al. observed upregulated expression of WTAP in nasopharyngeal carcinoma and found that it methylates DIAPH1-AS1 in a manner dependent on the m6A reader IGF2BP2, subsequently enhancing its stability. This, in turn, promotes the formation of the MTDH–LASP1 complex, leading to the upregulation of LASP1 expression and ultimately promoting the growth and metastasis of nasopharyngeal carcinoma (104). Regarding laryngeal carcinoma, the methyltransferase domain of YTHDF1 has been shown to interact with the 3′-UTR and 5′-UTR of TRFC mRNA, resulting in the promotion of its translation. Experimental validation further suggested that TFRC serves as a key target of YTHDF1-mediated increases in iron metabolism (105). These findings highlight the critical involvement of m6A RNA methylation in the progression and metastasis of respiratory system tumors. Understanding the molecular mechanisms underlying these processes could lead to the identification of potential therapeutic targets and strategies for treating aggressive malignancies.

### Digestive system

In recent years, numerous studies have highlighted the significant role of m6A modifications in the occurrence and development of digestive system tumors. Guo et al. identified heterogeneous nuclear ribonucleoprotein A2B1 (HNRNPA2B1) as an m6A reader that promotes the proliferation, migration, and invasion of esophageal cancer by upregulating expression of the fatty acid synthases ACLY and ACC1 (86). In esophageal squamous cell carcinoma, Wang et al. found that METTL3 enhances the m6A modification of *APC* and recruits YTHDF for *APC* mRNA degradation. In turn, decreased APC expression increases β-catenin and β-catenin-mediated cyclin D1, c-Myc, and PKM2 expression, leading to enhanced aerobic glycolysis in mice, which ultimately promotes cell proliferation and tumor formation (87).

In gastric cancer, WTAP, as an m6A methyltransferase, enhances the stability of *HK2* mRNA by binding the 3′-UTR m6A site, thereby promoting the proliferation and glycolysis of gastric cancer (88). In hepatocellular carcinoma, HBXIP drives the metabolic reprogramming and malignant biological behavior of cancer cells by positively regulating the METTL3-mediated m6A modification of *HIF-1α* (106). Moreover, Zuo et al. reported that in this malignancy, METTL3 regulates the m6A modification of LINC00958, enhancing its stability, and that LINC00958 ultimately promotes cancer cell adipogenesis by upregulating the expression of HDGF (90).

Guo et al. found that METTL3 stimulates the m6A modification of *DEGS2* and that YTHDF2 binds to the m6A site, promoting *DEGS2* decay. As a result, ceramide synthesis was found to be increased due to the downregulation of DEGS2 expression (107). Additionally, Shen et al. demonstrated that METTL3 stabilizes the expression of HK2 and GLUT1 in colorectal cancer through an m6A–IGF2BP2/3-dependent mechanism, thereby regulating glucose metabolism (91). Moreover, the N6-methyladenosine reader IMP2 activates the Warburg effect by stabilizing the ZFAS1/OLA1 axis (92).

### Reproductive system

Research on m6A modifications in reproductive system tumors, including cervical, ovarian, and prostate cancers, is ongoing. Wang et al. reported that METTL3 enhances the stability of *HK2* through YTHDF1-mediated m6A modifications, promoting the Warburg effect in cervical cancer (93). Additionally, the downregulation of E6/E7 and IGF2BP2 expression has been shown to decrease aerobic glycolysis capacity and the growth of cervical cancer cells. Mechanistically, the E6/E7 protein was found to regulate *MYC* mRNA m6A modifications through IGF2BP2, thus promoting cervical cancer cell aerobic glycolysis, proliferation, and metastasis. That study further highlighted the role of E6/E7 in promoting cervical cancer by activating IGF2BP2 and established a link with the promotion of aerobic glycolysis (95).

In ovarian cancer, HIF-1α has been found to regulate the overexpression of WTAP in an m6A-dependent manner, thereby promoting the Warburg effect in tumors. The underlying mechanism involves an interaction between WTAP and DGCR8 to regulate miR-200 expression in an m6A-dependent manner. Simultaneously, miR-200 positively regulates the key glycolytic enzyme HK2, ultimately affecting the intracellular Warburg effect (96).

Research by Liu et al. on prostate cancer revealed that METTL3 promotes the stability of lncRNA SNHG7 through an m6A modification, and this stable lncRNA accelerates glycolysis in prostate cancer through the SRSF1/c-Myc axis (97). Furthermore, ALKBH5 overexpression inhibits cervical squamous cell carcinoma growth and lipid metabolism both *in vivo* and *in vitro*. Mechanistically, ALKBH5 inhibits ACC1 deacetylation by promoting SIRT3 methylation, and the resulting downregulation of ACC1 expression ultimately inhibits tumor growth, lipid metabolism, and tumorigenesis (94).

### Urinary system

M6A modifications also play a significant role in the regulation of metabolism within the urinary system. Zhang et al. demonstrated that the downregulation of METTL14 expression in renal cell carcinoma might be linked to tumor development and metabolic regulation. Notably, METTL14 deficiency was found to promote the metastasis of renal cell carcinoma in both *in vivo* and *in vitro* experiments. Mechanistically, METTL14-mediated m6A modifications were shown to have a negative regulatory effect on the stability of bromine PHD finger transcription factor (*BPTF*) mRNA, with BPTF playing a pivotal role in pulmonary metastasis. BPTF functions as a super-enhancer that activates downstream targets, including enolase 2 and SRC proto-oncogene non-receptor tyrosine kinase, thereby leading to the glycolytic reprogramming of METTL14^−/−^ cells (98). Yuan et al. demonstrated that the m6A-modified reader IGF2BP1 promotes glycolysis, which involves glucose uptake, lactic acid production, and extracellular acidification. Mechanistically, IGF2BP1 recognizes the m6A modification site on *LDHA* mRNA, leading to enhanced mRNA stability and the consequent acceleration of tumor energy metabolism. Thus, m6A modulations appear to play a significant role in mediating aerobic glycolysis in clear-cell renal cell carcinoma (99). Furthermore, MTHFD2 expression was found to be significantly elevated in renal cell carcinoma, with sequencing data indicating that MTHFD2 can promote the translation of HIF-2α, thereby further enhancing aerobic glycolysis. Consequently, MTHFD2 establishes an association between the RNA methylation status in renal cell carcinoma and the metabolic state of tumor cells (100).

Expanding on our understanding of bladder cancer, ALKBH5 expression was found to be significantly downregulated, and its low expression is associated with poor prognosis in patients. Additionally, the downregulation of ALKBH5 expression promotes bladder cancer cell proliferation, migration, and invasion, resulting in reduced sensitivity to cisplatin chemotherapy. Mechanistically, ALKBH5 further regulates the casein kinase 2 (CK2) α-mediated glycolysis pathway by mediating the stability of CK2α, ultimately inhibiting bladder cancer progression in an m6A-dependent manner and increasing cisplatin sensitivity (101). Moreover, Liu et al. identified METTL14 as a primary m6A-related enzyme in bladder cancer. Mettl14-mediated m6A modifications were found to promote the expression of lncDBET, and the upregulation of lncDBET expression activates the PPAR signaling pathway, which, through a direct interaction with FABP5, promotes lipid metabolism in cancer cells, thus driving the malignant progression of BCa both *in vitro* and *in vivo* (102).

### Hematologic system

In addition to solid tumors, for hematological tumors, a series of studies have also reported the regulation of m6A methylation modification in metabolism.For example, the m6A reader IGF2BP2 modulates glutamine metabolism in acute myeloid leukemia (AML), and targeting IGF2BP2 could serve as a novel therapeutic strategy for the disease (108).Moreover, METTL16 drives the development of leukemia and the self-renewal of leukemia stem cells by reprogramming the metabolism of branched-chain amino acids. This metabolic reprogramming identifies METTL16 as a potential therapeutic target for combating leukemogenesis (109).m6A modification of circTET2, by interacting with HNRNPC, orchestrates fatty acid oxidation to enhance the proliferation of chronic lymphocytic leukemia. This mechanism highlights circTET2 as a novel therapeutic target for managing the disease (110).Furthermore, R-2-hydroxyglutarate dampens aerobic glycolysis in leukemia through the FTO/m6A/PFKP/LDHB signaling axis, offering a potential therapeutic approach for modulating leukemia metabolism (111).

### Other systems

In addition to the above studies, in our nervous system tumors, In addition, ALKBH5, FTO, YTHDF2 and other key enzymes that regulate m6A methylation play an indispensable regulatory role in the occurrence and development of glioma. Therefore, it may provide more diversified options for the treatment of glioma (112–114). However, there are limited reports on the regulation of m6A methylation modification on metabolism in gliomas.Ruan C et al’s research highlights the role of METTL3 in promoting aerobic glycolysis in glioma cells through the regulation of the m6A/miR-27b-3p/PDK1 signaling pathway, which is crucial for the tumorigenic metabolism and proliferation of glioma cells (115).Interestingly, a study by Zhang J et al. found cholesterol metabolism plays a critical role in glioma malignancy, with the hnRNPA2B1 protein acting as a key regulator. It demonstrates that hnRNPA2B1-dependent regulation of the sterol regulatory element-binding protein 2 (SREBP2) and the low-density lipoprotein receptor (LDLR) contributes to the dysregulation of cholesterol metabolism, thereby promoting the malignant characteristics of glioma cells (116). Meanwhile, another recent study showing the forced expression of malate dehydrogenase 2(MDH2) raised m6A levels and suppressed ALKBH5 activity in glioblastoma stem cells, which could be restored by alpha-ketoglutarate supplementation. Conversely, targeting MDH2 decreased overall m6A levels and affected the expression of platelet-derived growth factor receptor-β. Inhibiting MDH2 in glioblastoma stem cells enhanced the effectiveness of dasatinib, a multi-kinase inhibitor, suggesting a potential therapeutic approach for targeting stem-like tumor cells by reprogramming their metabolism (117).

These studies collectively demonstrate the crucial role of m6A modifications in the metabolic reprogramming and progression of various tumors. Further investigations in these areas hold great potential for advancing our understanding of tumor biology and developing novel therapeutic strategies.

## Role of m6A modifications in the tumor immune microenvironment

The TME refers to the surrounding environment in which tumor cells exist, including blood vessels, immune cells, fibroblasts, bone marrow-derived inflammatory cells, various signaling molecules, and the extracellular matrix (118). The main cellular components of the TME have a dual role in tumor progression, as they maintain an immunosuppressive microenvironment and have anti-tumor effects in the early stages. The aim of TME-targeted immunotherapeutic strategies is to stimulate or restore the innate tumor-suppressive ability of the immune system, reshaping a positive immune microenvironment and producing a comprehensive response (119).

**Figure 4:**
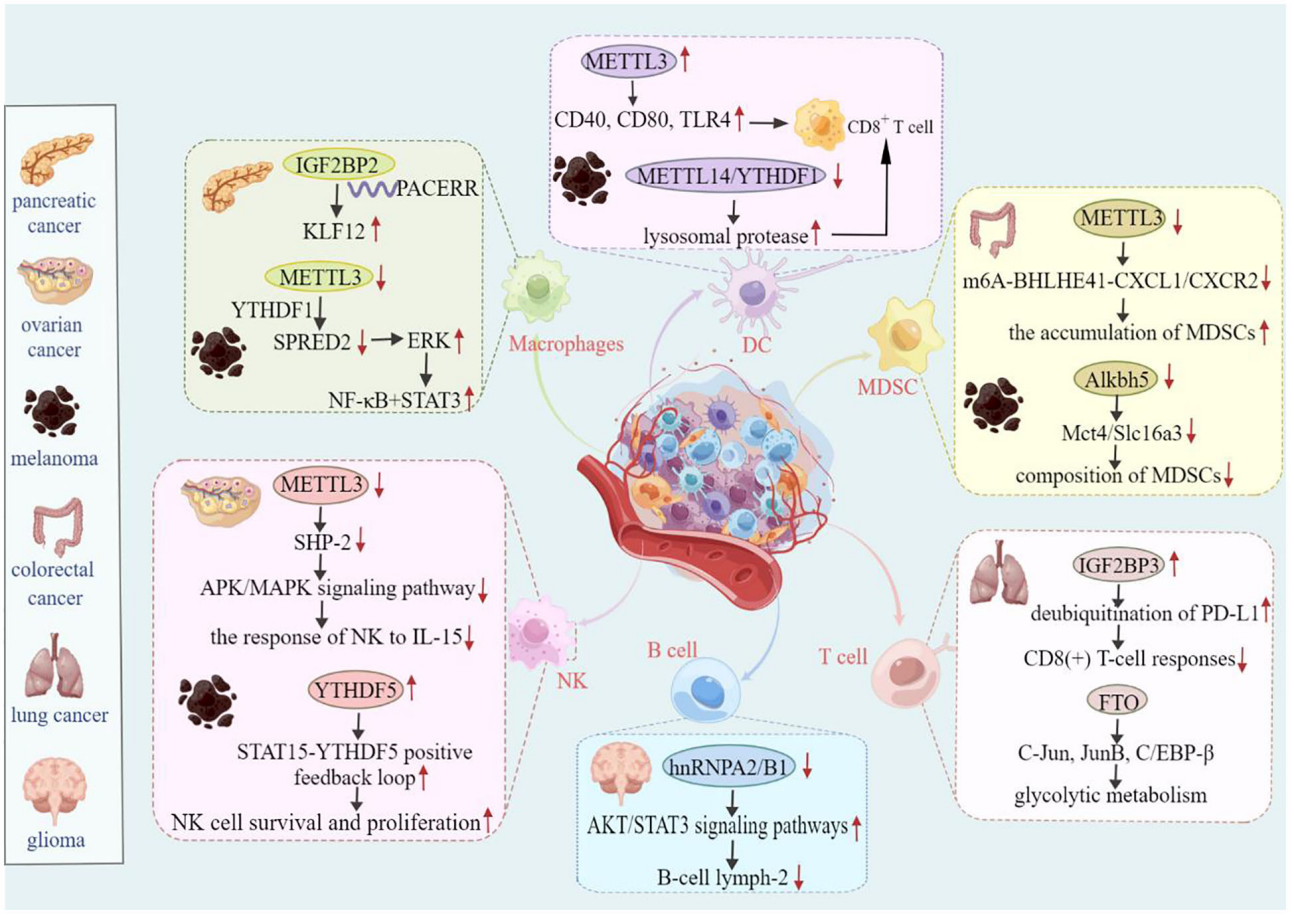
Mechanism of m6A-mediated tumor regulation in the immune microenvironment. For example, in ovarian cancer, METTL3 affects NK cell-mediated immunity by regulating the APK/MAPK signaling pathway. IGF2BP3 further influences the immune response of CD8-positive T cells in lung cancer by positively regulating deubiquitination of PD-L1. Therefore, m6A regulators are essential for the regulation of tumor immune effects.

It is well known that antigens on the surfaces of malignant tumors can be specifically recognized by host T cells. Additionally, various immune cell types, such as T cells, B cells, natural killer (NK) cells, Natural killer T (NKT) cells, basophils, neutrophils, DCs, and bone marrow-derived suppressor cells (MDSCs), are involved in the regulation of cancer progression (120, 121). Studies have shown that METTL3-deficient mice exhibit tumor infiltration by M1/M2-like tumor-associated macrophages and regulatory T cells (122). Moreover, several studies have reported that m6A modifications can regulate immune cell activation and the tumor immune microenvironment, ultimately affecting the efficacy of immunotherapy (123). In this section, we review the potential role of m6A modifications in immune regulation and the related regulatory mechanisms. The role of m6A modifications in the tumor immune microenvironment is summarized in **Table 2**, and the mechanism underlying the role of m6A in tumor regulation in the immune microenvironment is shown in **Figure 4**.

**Table 2:** Role of m6A modifications in the tumor immune microenvironment.

Cancer types	Immune cell	m6A regulators	Mechanisms	Ref.
Melanoma	Macrophages	METTL3↓	Facilitates M1 and M2 polarization through NF-kB/STAT3	(122)
PDAC	Macrophages	IGF2BP2↑	IGF2BP2 binds to LncRNA-PACERR to increase the number of M2-type polarized cells	(124)
Melanoma/CRC	DCs	YTHDF1↓	YTHDF1-deficient DCs can activate T cells	(125)
CRC	DCs	YTHDF1↓	Lack of YTHDF1 increases lysosomal protease production	(126)
Melanoma	NK cells	YTHDF5	STAT15–YTHDF5 positive feedback loop promotes NK cell functions	(127)
Melanoma	NK cells	FTO↑	FTO negatively regulates IL-2/15-driven JAK/STAT signaling by increasing the mRNA stability of SOCS	(128)
OV	NK cells	METTL3↓	Reducing HP-2 expression and the activation of AKT and MAPK signaling pathway	(129)
CRC	MDSCs	METTL3↑	METTL3 regulates the m6A–BHLHE41–CXCL1/CXCR2 axis	(130)
GBM	B cells	hnRNPA2/B1↓	Leads to inactivation of AKT and STAT3 signaling pathways	(131)
NSCLC	T cells	IGF2BP3↑	Promotes the deubiquitination of PD-L1	(132)

MDSCs, myeloid-derived suppressor cells; DCs, dendritic cells; NK, natural killer; PDAC, pancreatic ductal adenocarcinoma; CRC, colorectal cancer; OV, ovarian cancer; GBM, glioblastoma; NSCLC, non small cell lung cancer.

↑ indicates that increased expression of m6A regulators, and the ↓ indicates that decreased expression of m6A regulators.

### Macrophages

Macrophages are essential regulators of the immune system and include classically activated macrophages (M1) and alternatively activated macrophages (M2), the polarization of which influences the occurrence and development of various diseases. M1 macrophages can kill tumor cells and facilitate resistance to pathogen invasion, whereas M2 macrophages predominantly contribute to tumor growth, invasion, and metastasis (133). Tumor-associated macrophages (TAMs) comprise a prominent population of macrophages that infiltrate tumor tissues, representing the largest number of immune cells within the tumor microenvironment. TAMs secrete various cytokines and play a role in recognizing and clearing tumor cells in the early stages of tumor development. However, as tumors progress, they also play a significant role in promoting tumor growth, invasion, and metastasis, rendering TAMs a “double-edged sword” of tumor occurrence and development (134).

METTL3 depletion in macrophages reshapes the tumor microenvironment by enhancing M1- and M2-like TAM phenotypes (122). Liu et al. reported that in pancreatic ductal adenocarcinoma, lncRNA-PACERR increases the number of M2-type polarized cells and promotes cell proliferation, invasion, and migration both *in vivo* and *in vitro*. Mechanistically, IGF2BP2 enhances the cytoplasmic stability of KLF12 and c-Myc by binding to lncRNA-PACERR in an m6A-dependent manner (124). Cui et al. showed that METT13-deficient mice exhibit increases in M1/M2-like TAMs and regulatory T-cell infiltration compared to those in wild-type mice. Mechanistically, METTL3 loss affects YTHDF1-mediated SPRED2 translation, thereby enhancing NF-kB and STAT3 activation via the ERK pathway (122).

### Dendritic cells

DCs are primarily responsible for antigen processing and presentation and the activation of T cell immune responses (135). These cells play different roles at various stages of maturation. For example, immature DCs induce immune tolerance, mature DCs activate and stimulate immune responses, and regulatory DCs suppress immune responses by inhibiting T-cell responses (136).

m6A modifications play a crucial role in tumor immunomodulation, particularly in immune regulation involving DCs. For example, the methyltransferase METTL3 promotes DC activation and function. Mechanistically, the METTL3-mediated m6A modification of the CD40, CD80, and TLR4 signaling adaptor *Tirap* transcripts enhances their translation in DCs, thereby stimulating T-cell activation and enhancing TLR4/NF-κB signaling-induced cytokine production (137). The knockout of YTHDF1 in DCs enhances the cross-presentation of tumor antigens *in vivo* and the cross-activation of CD8^+^ T cells (138). In YTHDF1-deficient melanoma or colon cancer, antigen cross-presentation by tumor-infiltrating DCs induces a stronger anti-cancer immune response and mature YTHDF1-deficient DCs can activate T cells more effectively than wild-type cells, leading to stronger anti-tumor immunity (125). A separate study also demonstrated that mice lacking YTHDF1 exhibit an elevated and more effective CD8^+^ T-cell response against tumor-specific antigens. This phenomenon was attributed to a specific mechanism involving RNA methylation (m6A modification), in which transcripts encoding lysosomal proteases are marked. YTHDF1 was further found to bind to these modified transcripts, subsequently leading to the increased translation of lysosomal pepsin within DCs. Interestingly, the inhibition of cathepsin, a protein involved in cellular processes, notably heightens the cross-presentation ability of normal DCs. Furthermore, the positive effect of blocking the PD-L1 checkpoint pathway was found to be notably enhanced in mice lacking the *YTHDF1* gene (YTHDF1^−/−^ mice). This observation suggests that YTHDF1 might serve as a promising target for enhancing the effectiveness of anti-cancer immunotherapies (126).

DC-specific lnc-Dpf3 facilitates chemokine receptor 7 (CCR7)-mediated DC migration, leading to an excessive adaptive immune response and inflammatory damage. Mechanistically, YTHDF2 reduces the expression level of lnc-Dpf3 in resting mature DCs. However, CCR7 can upregulate the expression of lnc-Dpf3 by removing m6A modifications, thus stimulating the rapid but transient migration of DCs to the draining lymph nodes (139). Therefore, further exploration of the crucial regulatory role of m6A in dendritic cells could lead to innovative anti-tumor approaches.

### Natural killer cells

NK cells are the cornerstone of natural immunity and constitute approximately 5–15% of the lymphocyte population. They possess the unique ability to promptly and directly eliminate foreign entities, such as virus-infected cells, cancer cells, and senescent cells, without the need for antigen presentation (140). Ma et al. revealed the importance of YTHDF2 in NK cells, showing that its deficiency can compromise their *in vivo* anti-tumor and antiviral activities. The underlying mechanism was determined to involve YTHDF2 promoting NK cell functions through the formation of a STAT15–YTHDF5 positive feedback loop, which is crucial for IL-2-mediated NK cell survival and proliferation (127). Moreover, m6A methylation plays a protective role in NK cell homeostasis and tumor immune surveillance. METTL3 in NK cells is essential for maintaining homeostasis, as it promotes the infiltration and functionality of NK cells in the TME, thereby enhancing anti-tumor immunity and increasing overall survival in mice (129). In addition, FTO serves as a significant regulator of NK cell immunity. *In vivo* studies have shown that FTO knockout in mouse NK cells prevents melanoma metastasis, whereas *in vitro* experiments have indicated that FTO-deficient human NK cells exhibit an enhanced anti-tumor response to leukemia, thereby providing a promising strategy for allogeneic NK cell therapy (128). In ovarian carcinoma, the depletion of METTL3 in NK cells inhibits cell infiltration ability and functions, leading to accelerated tumor development through a reduction in SHP-2 expression and AKT and MAPK signaling pathway activation (129). Therefore, the modulation of NK cells by modifying m6A methylation could be a promising anti-cancer therapeutic strategy.

### Myeloid-derived suppressor cells

MDSCs are a heterogeneous group of myeloid cells with immunosuppressive activity that have been identified as potential precursors of DCs, macrophages, and granulocytes (141). Within the TME, MDSCs play a critical role in suppressing immune cells and protecting tumors, leading to immunotherapy resistance and tumor progression, ultimately contributing to patient mortality (141). As such, targeting and eliminating MDSCs have emerged as promising strategies to improve response rates to cancer treatments and enhance patient survival (142).

Chen et al. showed that METTL3 enhances the expression of BHLHE41 by facilitating m6A modifications, subsequently leading to the increased transcription of *CXCL1* and *CXCR2*. This enhances the migration and accumulation of MDSCs in the tumor microenvironment, ultimately suppressing the activation and proliferation of CD8+ T cells and other immune cells. The suppression of anti-tumor immunity by METTL3 through the m6A–BHLHE41–CXCL1/CXCR2 axis contributes to tumor immune evasion and progression (130). Targeting YTHDF1 enhances the anti-tumor immune response and increases the effectiveness of anti-PD-1 therapy, providing a potential novel strategy for the treatment of colorectal cancer (143). The inhibition of Alkbh5 was found to enhance the efficacy of cancer immunotherapy. Alkbh5 was also shown to regulate the expression of target genes in the TME and the composition of MDSCs, which can influence the response to immunotherapy (144). Furthermore, the knockout of METTL3 in CD33^+^ cells attenuates MDSC differentiation and the generation of tumor-associated MDSCs *in vitro* (145). Overall, targeting MDSCs through m6A methylation modification appears to have significant potential for improving the efficacy of anti-tumor therapy. These studies provide an understanding of the intricate regulatory role of m6A RNA methylation in the modulation of MDSCs and its effect on tumor immunity.

### B cells

B cells play a central role in humoral immunity, primarily by secreting antibodies, presenting soluble antigens, and regulating immune responses. This section provides a comprehensive review of the involvement of the three major m6A RNA modifiers in B-cell development and B cell-related diseases. The germinal center (GC) is responsible for generating protective antibodies with long-lasting immune effects. Grenov et al. demonstrated that YTHDF2-mediated m6A modifications are crucial for GC maintenance. In the absence of YTHDF2, the expression of genes related to cell proliferation and oxidative phosphorylation in GC B cells is reduced, resulting in slowed cell cycle progression (146). Interleukin-7 (IL-7) induces B cell transformation, and Mettl14 is essential for this process. Accordingly, its deficiency affects B-cell development and leads to abnormalities in critical gene expression programs during B-cell maturation (147). Additionally, Yin et al. showed that the downregulation of hnRNPA2/B1 expression can lead to the inactivation of the AKT and STAT3 signaling pathways, which subsequently reduces the expression of B cell lymph-2 (Bcl-2), ultimately inhibiting the growth of glioma. These findings highlight the potential of hnRNPA2/B1 as a therapeutic target for glioma treatment (131).

Overall, this research emphasizes the importance of m6A RNA modifications in B-cell biology. It further highlights the potential of targeting B cells for immune regulation as a promising direction for anti-tumor therapy. Understanding the molecular mechanisms involved in m6A RNA modifications with respect to B-cell development and functions might thus provide valuable insights for the development of novel immunotherapeutic strategies against B cell-related diseases and cancer.

### T cells

T cells, also known as T lymphocytes, are derived from lymphocyte stem cells in the bone marrow and undergo differentiation and maturation in the thymus. They are distributed to various immune organs and tissues throughout the body via the lymph and blood circulation systems, where they perform critical immune functions (148). In both *in vitro* and *in vivo* studies, the inhibition of METTL3 or IGF2BP3 was shown to enhance anti-tumor immunity through the PD-L1-mediated activation, depletion, and infiltration of T cells (149). In NSCLC, m6A-modified circIGF2BP3 promotes the deubiquitination of PD-L1, leading to immune evasion and resistance to treatment utilizing CD8^+^ T cells (132). The loss of METTL3 also disrupts T-cell homeostasis and differentiation, as observed by Li et al. (150). Furthermore, regulation of the m6A methyltransferase Mettl14 in TAMs can promote CD8^+^ T cell dysfunction and tumor progression (151). Methionine metabolism-derived S-adenosylmethionine promotes m6A methylation and the translation of immune checkpoints, including PD-L1 and the V-domain Ig suppressor of T cell activation (VISTA), in tumor cells. For example, the depletion of MRD or the m6A-specific binding protein YTHDF1 inhibits tumor growth by restoring CD8^+^ T-cell infiltration and, when combined with PD-1 blockade, this results in improved tumor control (152).

Overall, these findings emphasize the crucial role of m6A RNA modifications in regulating T-cell functions and immune responses in various cancers. Understanding the complex interactions between m6A modifiers and immune checkpoints will provide valuable insights into the development of new immunotherapeutic strategies for cancer treatment. However, further research is required to fully explore the potential of m6A RNA modifications as targets for cancer immunotherapy.

## Regulation of metabolic reprogramming by m6A in tumor immunotherapy

Based on the aforementioned studies and reports, many researchers have designed specific immune subgroups and molecular therapies for immunotherapy. Zegui et al. reported that m6A is enriched in the 3′-UTR of PD-L1 mRNA, corresponding to the immune checkpoint, and that the JNK signaling pathway promotes immune escape in bladder cancer by regulating m6A modifications of PD-L1 mRNA, mediated by METTL3 (153). Yi et al. reported that FTO, as an m6A demethylase, increases expression of the transcription factors C-Jun, JunB, and C/EBP-β and regulates glycolytic metabolism to evade tumor immune surveillance. Therefore, they developed a small-molecule compound, called Dac51, which further disrupts glycolysis in tumor cells by inhibiting FTO activity. Consequently, the anti-tumor effect of CD8^+^ T cells was determined to be restored, ultimately inhibiting tumor growth (154).

Su et al. developed two highly potent *in vitro* anti-leukemia FTO inhibitors, CS1 and CS2, which limit the self-renewal of cancer stem cells, increasing the sensitivity of leukemia cells to T-cell toxicity and overcoming hypomethylated drug-induced immune evasion (155). Moreover, Liu et al. concluded that circQSOX1 modified with m6A could promote immune escape in colorectal cancer by activating glycolysis and inactivating the anti-CTLA-4 therapeutic response. Therefore, the combination of sh-circQSOX1 and anti-CTLA-4 therapy is expected to provide a new direction for treating Treg cell-mediated immunotherapy resistance in colorectal cancer (156). The loss of METTL3 inhibits YTHDF1-mediated SPRED2 translation and further enhances the activation of NF-kB and STAT3 through the ERK pathway, ultimately mediating tumor growth and metastasis. In addition, the inhibition of PD-1 checkpoints results in a diminished therapeutic effect in METTL3-deficient mice, indicating that METTL3 could serve as a potential target for tumor immunotherapy (122). In breast cancer cells, PD-L1 is a downstream target of METTL3-mediated m6A modifications. This suggests that METTL3 upregulates the expression of PD-L1 through m6A–IGF2BP3-dependent transcription, further promoting the stability of PD-L1 mRNA. Therefore, the inhibition of METTL3 might be a new target for anti-tumor immunotherapy directed at PD-L1 (149). Juan et al. discovered a novel circular RNA, circRHBDD1, which not only promotes glycolysis but also inhibits the efficacy of anti-PD-1 therapy in liver cancer. This mechanism involves circRHBDD1 recruiting the m6A reader YTHDF1 to *PIK3R1* mRNA, ultimately accelerating the translation of PIK3R1. That study suggests that the inhibition of circRHBDD1 can enhance the efficacy of anti-PD-1 therapy for the treatment of hepatocellular carcinoma (157). Yin et al. concluded that the absence of METTL3 in macrophages weakens the efficacy of PD-1 blockade during the treatment of B16 tumors (122). Moreover, Chen et al. discovered that non-SMC lectin I complex subunit H (NCAPH) exhibits a strong association with the glycolytic activity and immune tolerance in clear-cell renal cell carcinoma. Throughout its expression, the stability and nuclear export of NCAPH mRNA are regulated by IGF2BP3 and YTHDC1 in an m6A-dependent manner. Elevated NCAPH expression promotes cancer cell glycolysis, enhances PD-L1 expression, and induces resistance to anti-PD-1 therapy by stabilizing the β-catenin protein (158). Furthermore, GNRa-CSP12 has emerged as a promising immunosuppressive agent for leukemia treatment. Its mechanism involves disruption of the intracellular Fe^2+^/Fe^3+^ balance, leading to the inactivation of the Fe^2+^-dependent demethylases FTO and ALKBH5. Consequently, the intracellular m6A levels increase. This elevation in m6A can detrimentally affect the stability of the transcripts of key molecules involved in glycolysis (GLUT3) and the immune checkpoint pathway (PKM). By destabilizing GLUT3 and PKM transcripts, GNRa-CSP12 effectively inhibits the proliferation of AML cells and enhances the therapeutic efficacy of PD-L1 checkpoint blockade (159). The regulation of m6A on metabolic reprogramming in tumor immunotherapy is shown in **Table 3**.

**Table 3:** Regulation of metabolic reprogramming by m6A in tumor immunotherapy.

Cancer types	m6A regulators	Immune checkpoint	Metabolism	Mechanisms	Ref.
CRC	FTO	PD-1	Glycolysis	FTO increases the transcription factors C-Jun, JunB, and C/EBP-β	(154)
HCC	YTHDF1	PD-1	Glycolysis	circRHBDD1 recruits the m6A reader YTHDF1 to PIK3R1 mRNA	(157)
RCC	IGF2BP3 YTHDC1	PD-1	Glycolysis	High expression of NCAPH promotes tumor glycolysis and induces resistance to anti-PD-1 therapy by stabilizing the β-catenin protein	(158)
CRC	METTL3	CTLA-4	Glycolysis	m6A-modified circQSOX1 can recruit miR-326 and miR-330-5p, enhance glycolysis, and reduce the therapeutic effect of anti-CTLA-4 agents	(156)

CRC, colorectal cancer; HCC, hepatocellular carcinoma; RCC, renal cell carcinoma.

In summary, m6A modifications play a pivotal role in the regulation of tumor metabolism, particularly glycolysis. Understanding the intricate relationship between m6A modifications and metabolic pathways has great potential for tumor immunotherapy. For example, exploring the synergistic effects of immune checkpoint inhibitors represents a promising direction for future research aimed at improving anti-tumor therapies.

## Conclusions and perspectives

The dysregulation of m6A regulators occurs across various stages of tumorigenesis and exerts notable effects on processes such as metabolism, metastasis, proliferation, drug resistance, and immunotherapy. Aberrant metabolic processes can promote tumor growth, while concurrently contributing to malignant progression. Hence, understanding the involvement of m6A modifiers in metabolic regulation and their interplay within the immune microenvironment is paramount for preventing tumorigenesis.

Within the tumor immune microenvironment, investigating the synergistic effects between m6A modifiers and key metabolic enzymes or the dual inhibition of both modifiers and metabolic enzymes has emerged as a promising avenue. Several pivotal breakthroughs have significantly enriched our understanding of m6A-dependent immune modulation, thereby forming a basis for future translational advancements in m6A-oriented immunotherapies. An illustrative example involves inducing YTHDF1 deficiency in DCs via checkpoint blockade, as a potential immunotherapeutic strategy (126).

Nevertheless, the roles and mechanisms of m6A methylation in tumors extend beyond metabolic regulation and immune microenvironmental influences. In recent years, there has been an upsurge in the investigation of m6A-mediated tumor drug resistance. For example, Zhuang et al. highlighted the involvement of m6A methylation in diverse processes, including RNA stability, splicing, transcription, translation, and degradation. Importantly, it was also found to have a pivotal role in the emergence of resistance to cancer treatments, including chemotherapy, immunotherapy, and radiotherapy. These phenomena stem from the capacity of the drug to modulate the expression of specific m6A-related genes, thereby directly affecting methylation levels (a direct effect). Furthermore, drugs can act directly on target genes, subsequently regulating downstream m6A-associated genes, and inducing alterations in m6A methylation levels (indirect effects) (160). Consequently, the manipulation of m6A regulation can exert discernible effects on the efficacy of tumor therapies.

Recent studies have attempted to design tailored inhibitors that target m6A methylation. Notably, Yankova et al. elucidated the potential of STM2457, a small-molecule inhibitor targeting METTL3, to suppress AML. These findings underscore the potential therapeutic utility of METTL3 inhibition, thereby establishing a significant therapeutic paradigm for RNA-modifying enzymes (161). Similarly, studies have highlighted the role of ALK-04 in suppressing Treg and MDSC infiltration via ALKBH5 inhibition, thus augmenting the effectiveness of anti-PD-1 therapy (121). Several studies have further confirmed the inhibitory effects of rhein on FTO. Rhein predominantly competes for the active site of FTO *in vitro*, demonstrating robust inhibitory activity against m6A demethylation within cells (162). The strategic design of inhibitors targeting pivotal m6A modification enzymes facilitates the precise regulation of methylation patterns, thereby exerting a discernible effect on tumor progression.

Given the multifaceted mechanisms through which m6A modifiers orchestrate tumor dynamics, the development of inhibitors targeting these modifiers has tremendous therapeutic potential against tumors. Although certain studies have elucidated the inhibitory effects of corresponding modifiers, the translation of these findings into clinical therapies remains an avenue for exploration. Further investigation of the intricate mechanisms underlying m6A modifier-mediated metabolic regulation within the tumor immune microenvironment promises to yield novel directions and renewed optimism for contemporary anti-tumor therapeutic strategies.
